# Attentional Cueing Modifies the Observed Association Between Post-Set Lactate and Velocity Loss During Smith Machine Bench Press

**DOI:** 10.3390/jfmk11020189

**Published:** 2026-05-11

**Authors:** Fernando Martin-Rivera, Darío Rodrigo-Mallorca, Alvaro Juesas, Angel Saez-Berlanga, Iván Chulvi-Medrano

**Affiliations:** 1 Department of Physical Education and Sports, University of Valencia, 46010 Valencia, Spain; dariorodrigom@gmail.com (D.R.-M.); angel.saez@uv.es (A.S.-B.); ivan.chulvi@uv.es (I.C.-M.); 2 Research Group in Prevention and Health in Exercise and Sport (PHES), University of Valencia, 46010 Valencia, Spain; alvaro.juesastorres@uchceu.es; 3 Department of Education Sciences, CEU Cardenal Herrera University, 46115 Castellón, Spain

**Keywords:** internal focus, external focus, barbell speed, mechanical fatigue, capillary blood sampling

## Abstract

**Background:** Velocity loss (VL) is widely used in velocity-based training (VBT) to index mechanical fatigue, yet attentional focus cues may alter velocity profiles and their relationship with internal load. This study tested whether internal focus, external focus, or control modifies repetition-level velocity, lactate kinetics, and lactate–VL% coupling during bench press (BP) at 60% one-repetition maximum (1RM). **Methods**: Thirty-six trained men were randomized into three groups. Thirty-four participants completed the study and were included in the final analyses according to outcome-specific data availability. Participants completed two counterbalanced sessions on a Smith machine BP: (i) a single set to technical failure, and (ii) a conventional 3 sets × 10 repetitions at 60% 1RM. Concentric velocity was recorded via a linear position transducer and analyzed at the repetition level using linear mixed-effects models. Lactate was analyzed via Gaussian generalized estimating equations (GEEs). **Results**: Repetitions to failure and terminal velocity at failure did not differ between groups (Welch *p* = 0.328; *ω*^2^ = 0.045). During 3 sets × 10 repetitions, velocity decreased across sets and repetitions (both *p* < 0.001); adding group terms improved fit (LR χ^2^(12) = 42.26, *p* < 0.001), with additional improvement for group-dependent fatigue patterns (LR χ^2^(6) = 14.90, *p* = 0.021). Lactate increased over time (Wald χ^2^(4) = 244.56, *p* < 0.001) with convergence by post-lactate set 3 and post-lactate 30 s. Lactate–VL% coupling was strongly moderated by group (post-lactate × group: χ^2^(2) = 80.42, *p* < 0.001), with slopes (ΔVL% per 1 mmol·L^−1^) of 5.27 (internal focus), 13.60 (external focus), and 0.04 (control). After Holm correction across prespecified primary outcomes, only the post-session rating of perceived exertion differed (*p*Holm = 0.004; *ω*^2^ = 0.045), with higher values in the external focus group. Pairwise effects were calculated as comparator minus external focus; therefore, negative *g* values indicate a higher rate of perceived exertion (RPE) in the external focus group (*g*Hedges ≈ −1.50 vs. control; −1.37 vs. internal focus). **Conclusions**: Attentional cueing did not consistently alter averaged VL% outcomes after multiplicity correction, but it was associated with differences in early lactate kinetics and modified the observed association between post-set lactate and VL% in the interaction-based coupling model. Cueing scripts should therefore be reported verbatim and standardized in VBT studies, particularly when VL-derived indices are interpreted alongside internal load markers.

## 1. Introduction

Resistance training (RT) is widely regarded as a core strategy to enhance physical performance and promote health, and its effective prescription requires precise control of key dose components—namely intensity, volume, and proximity to failure—alongside consideration of the acute fatigue elicited by each session [1,2]. In this context, velocity-based training (VBT) has gained substantial traction because it enables real-time monitoring of the mechanical expression of effort on a repetition-by-repetition basis [3]. Within-set velocity loss (VL) has been advanced as a practical indicator of mechanical fatigue and a volume-regulation criterion, given that manipulating VL cut-offs (e.g., 10–30%) induces differentiated acute effects on performance outcomes and execution characteristics [4]. This rationale has been synthesized in a recent systematic review focusing on VL threshold implementation for effort management and internal load regulation [5], and is further corroborated by experimental work directly contrasting multiple VL thresholds in multi-joint movements [6].

Parallel to the evolution of VBT, evidence from the motor learning and movement control literature indicates that attentional focus instructions (internal vs. external) meaningfully modulate motor performance and execution efficiency. Broadly, directing attention toward the intended movement effect (external focus) is typically linked to more automatic control mechanisms, whereas focusing on body segments or muscle actions (internal focus) tends to increase the conscious regulation of movement, with potential consequences for efficiency and fatigue development during RT [7]. In strength-related tasks, attentional focus manipulations have been shown to influence both mechanical outputs and neuromuscular activation profiles, highlighting that the instructional set represents a relevant methodological and applied factor rather than a trivial procedural detail [8]. Moreover, attentional focus strategies have been associated with differences in perceived exertion and fatigue, suggesting that verbal cues may alter not only external performance but also the internal load experienced during RT [9,10].

When considered in the context of resistance exercises such as the bench press (BP), these findings have clear methodological relevance. Specifically, manipulating verbal instructions has been shown to alter electromyographic activity during execution, suggesting meaningful changes in motor strategy even when the external load is held constant [11]. Moreover, adopting an external attentional focus has been linked to improved performance in muscular endurance tasks, particularly under submaximal loads where the accumulation of fatigue may magnify subtle differences in movement coordination [12]. However, it remains uncertain whether such attentional focus-driven effects can be detected using kinematic metrics typically employed in VBT—such as velocity loss percentage (VL%) and the repetition-by-repetition decline slope—when participants are simultaneously instructed to perform the concentric phase with maximal intended velocity.

Within this framework, combining VBT and attentional focus manipulation in a single experimental design—while also including a physiological marker of internal load—can be considered as methodologically warranted. Available evidence indicates that VL thresholds not only shape training volume and the extent of mechanical fatigue, but are also accompanied by acute changes in internal markers such as blood lactate and rate of perceived exertion (RPE), highlighting the need to interrogate the coupling between mechanical degradation (e.g., VL) and metabolic strain [5,13]. Accordingly, if attentional focus influences execution efficiency [7] or alters the effective effort attained within a set [14], it is plausible that it could modify not only the velocity-time profile but also the relationship between VL and lactate kinetics during submaximal, high-repetition RT.

Accordingly, this study aimed to examine whether different attentional focus conditions (internal focus, external focus, and control) modulate the VBT profile during Smith machine BP performed at 60% of one-repetition maximum (1RM), while participants were instructed to execute the concentric phase with maximal intended velocity. In addition, we evaluated whether lactate kinetics and their relationship with VL vary across instructional conditions. We hypothesized that attentional focus could alter the development of mechanical fatigue—captured by indices such as VL% and the repetition-by-repetition decline slope—together with the associated metabolic cost. These outcomes have methodological relevance for cue standardization and for interpreting training dose when VL is used as a primary VBT criterion.

## 2. Materials and Methods

### 2.1. Study Design

**Figure 1:**
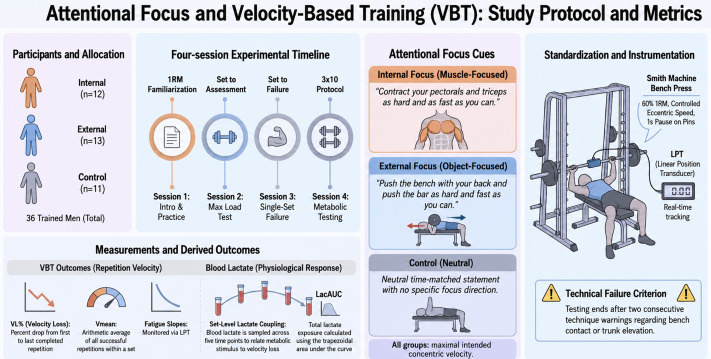
Overview of the experimental timeline, attentional focus conditions, exercise standardization, and derived velocity-based training and lactate outcomes. Note: VBT, velocity-based training; LPT, linear position transducer; AUC, area under curve; 1RM, one-repetition maximum.

A randomized, parallel group design was implemented to evaluate the influence of attentional focus instructions on VBT metrics and blood lactate kinetics during Smith machine BP. Participants were randomly allocated to one of three instructional conditions: internal focus, external focus, or control. The study comprised four sessions: (i) familiarization, (ii) 1RM assessment to prescribe 60% 1RM for subsequent trial, and two experimental sessions performed on different days: (iii) a single set to failure at 60% 1RM, and (iv) a conventional protocol consisting of 3 sets × 10 repetitions at 60% 1RM with repeated lactate sampling. The order of the two experimental sessions was counterbalanced to reduce potential sequencing effects. Sessions were separated by 48 h to limit residual fatigue and carry-over, and were scheduled at a consistent time of day (±1 h) for each participant under comparable environmental conditions to enhance methodological standardization. Primary outcomes included repetition-by-repetition bar velocity and derived VBT indices (e.g., within-set VL), alongside lactate time–course parameters and their relationships with VBT-derived fatigue markers (See Figure 1 for more detail information).

### 2.2. Participants

Thirty-six healthy adult men [mean (95% CI) ± SD: age 26.5 (24.2–28.8) ± 6.7 years; weight 78.7 (73.7–83.8) ± 14.5 kg; height 176.8 (174.7–178.9) ± 6.0 cm; body fat 17.8 (15.6–20.1) ± 6.5%; BP 1RM 102.2 (93.6–110.7) ± 25.1 kg; BP 1RM velocity 0.160 (0.143–0.176) ± 0.049 m·s^−1^; relative strength 1.29 (1.20–1.38) ± 0.26; 60% RM 61.3 (56.2–66.4) ± 15.1 kg] volunteered to participate. Participants were physically active sport science students with at least one year of RT experience (1–3 sessions per week) and were accustomed to executing BP with appropriate technique. No participant reported health-related limitations, musculoskeletal injuries or medical conditions that could compromise testing performance. Additionally, none were using medications, pharmacological treatments, or ergogenic substances expected to affect physical performance. The study was conducted in accordance with the Declaration of Helsinki and was approved by the Research Ethics Committee of the University of Valencia (reference: H1421157445503). Before participation, all participants received detailed information about the study aims and procedures and provided written informed consent.

### 2.3. Randomization and Blinding

Participants were randomly assigned in a 1:1:1 ratio to the internal focus, external focus, or control group using a computer-generated allocation sequence. Concealment of allocation was ensured through a secure digital platform accessible exclusively to the principal investigator. Participant blinding was not possible given the instruction-based nature of the intervention. To reduce potential bias, the researcher responsible for data processing and the statistician remained fully blinded to group identity; all datasets were anonymized and labeled as G1–G3 during preprocessing and model estimation, and the allocation key was withheld until the completion of the analyses.

### 2.4. Equipment and Exercise Standardization

Anthropometric outcomes were obtained using a medical stadiometer (Seca T214, Hamburg, Germany; precision 0.01 cm) and a bioelectrical impedance bascule (Tanita BF–350, Tokyo, Japan; precision 0.01 kg). All exercise testing was conducted on a Smith machine (Multipower Powerline PSM144X; Powerline, Forest Park, IL, USA). Barbell kinematics (displacement and velocity) were assessed with an iso-inertial dynamometer (Speed4Lift; Sped4Lift, Madrid, Spain) based on a cable-extension linear position transducer (LPT) affixed to the bar. Displacement was sampled at 1000 Hz and velocity was derived by time differentiation of the displacement signal [15], with data transmitted via Wi-Fi to an Android smartphone running the Speed4Lift application (v4.1; Speed4Lift, Madrid, Spain). Capillary blood samples were collected and analyzed using a portable lactate analyzer (Lactate Scout+; SensLab GmbH, Leipzig, Germany).

BP assessments were performed in accordance with a previously published procedure [16]. Participants adopted a supine position on a flat bench with the feet firmly placed on the floor, and grasped the bar with a grip slightly wider (2–3 cm) than biacromial width. The bench was individualized so that the bar’s vertical projection aligned with the participant’s intermammillary line. Strict technique was enforced: participants were instructed not to rebound the bar from the chest and to void lifting the shoulders or trunk during the lift. To standardize the eccentric range of motion across sets and to impose a controlled transition between phases, mechanical stops were positioned bilaterally on the Smith machine. These were adjusted so that the barbell stopped approximately 1 cm above the chest. After lowering the bar at a controlled mean eccentric velocity (~0.30–0.50 m·s^−1^), participants paused for ~1.0 s on the stops—momentarily unloading the load while maintaining hand contact—before initiating a purely concentric press executed with maximal intended velocity. This pause was implemented to reduce the influence of elastic rebound and to enhance measurement reliability and consistency [17]. While the eccentric phase was standardized by velocity control, the concentric phase was always performed with maximal intent. Individual setup parameters (bench position and grip width) were documented to ensure reproducibility across all testing sessions.

### 2.5. Session Procedures

Participants completed four monitoring sessions (≥48 h between sessions): (i) a familiarization session, (ii) a session to determine the actual external load corresponding to 60% 1RM, and two experimental sessions: (iii) a single set-to-failure test at 60% 1RM, and (iv) a conventional protocol consisting on 3 sets × 10 repetitions at 60% 1RM. Anthropometric measurements were collected during the familiarization session. During this session, participants were acclimated to the Smith machine BP setup, the bar velocity assessment system, and the capillary lactate sampling procedures. Standardized technique and safety instructions [18], together with the requirement to execute the concentric phase with maximal intended velocity, were explained and monitored under the supervision of a strength training professional (≥5 years of experience).

To establish the true 1RM—and, consequently, the individualized 60% 1RM load—participants performed a velocity-guided incremental BP test. Each session began with a standardized general warm-up consisting of 5 min of cycling or elliptical exercise followed by two sets of 10 push-ups separated by 2 min of rest. The BP protocol began at 20 kg, with sets of three repetitions. The load was increased by 10–20 kg between successive three-repetition sets until the maximum concentric velocity (Vmax) of the best repetition in a set dropped below 1 m·s^−1^; thereafter, sets were reduced to two repetitions and increments were lowered to 5–10 kg. Once the Vmax of the best repetition fell below 0.5 m·s^−1^, single-repetition sets were used and load increases were further reduced to 2.5–10 kg. As participants approached velocities typically observed near 1RM (~0.18 m·s^−1^), the smallest practicable increment (2.5–5 kg) was applied to enhance precision. Rest intervals were standardized at 3 min and were extended when required based on participant feedback to ensure adequate recovery before subsequent attempts. Testing was finalized when the participant failed to successfully complete a single repetition. Because fractional plates were unavailable, the minimum increment was 2.5 kg; if failure occurred after a 5 kg increase relative to the last successful lift, an intermediate load between the last successful and failed attempts was subsequently tested after full recovery to refine the 1RM estimate. Bar velocity was recorded for all attempts to inform objective decisions regarding repetition allocation and load progression.

During the first experimental session, participants performed a single set to volitional failure at 60% 1RM. Before this test set, the standardized general warm-up described previously was performed. In this protocol, failure was defined operationally as technical failure. Two investigators continuously supervised execution using predefined criteria, and a “warning” was issued when loss of shoulder and/or gluteal contact with the bench and/or trunk elevation was observed. Before data collection, both investigators were familiarized with these criteria to standardize their application. The set was finalized once the participant received two consecutive warnings for compromised technique. When uncertainty arose regarding a warning of termination decision, the investigators reached immediate consensus before ending the set, prioritizing participant safety and consistency of the stopping rule. Assistance was kept to a minimum and was provided only when necessary to ensure safety (i.e., if the participant could not complete the lift). The set was recorded to derive repetitions to failure, within-set VL, and the terminal velocity reached at the point of failure.

During the second experimental session, participants performed three sets of 10 repetitions at 60% 1RM, with 1 min of inter-set recovery. The sequence of the two experimental sessions was counterbalanced to minimize order effects. The same standardized general warm-up was performed. Rest periods were controlled with a stopwatch and fixed at exactly 60 s from the end of one set to the start of the subsequent set. If a participant reached failure before completing the prescribed repetitions, the set was finalized at that point. As in the set-to-failure session, finalization criteria were based on technical failure, defined as two consecutive technique warnings, and assistance was limited to situations required for safety. Blood lactate concentration was assessed at five time points: (i) baseline before warm-up; (ii) immediately after set 1; (iii) immediately after set 2; (iv) immediately after set 3; (v) 30 s after completion of the final set. To standardize the timing of the post-set samples, lactate was collected at a fixed interval of approximately 15 s after each set, once the participant had left the bench and sat upright. For the third set, this sample was obtained within a maximum of 15 s after exercise cessation to allow the subsequent 30 s post-exercise sample to be initiated accurately. This time window was selected to ensure procedural consistency and is compatible with previous evidence indicating that blood lactate sampling between 15 and 45 s can provide acceptable monitoring consistency [19]. Capillary blood was obtained from the earlobe, a commonly used sampling site [20], after cleansing and disinfection with 70% ethanol. A sample (≥0.5 μL) was then collected and analyzed using a portable lactate analyzer.

### 2.6. Attentional Focus

Attentional focus cues were administered to all participants by the same researcher. Across conditions, participants received identical instructions to execute the concentric phase with maximal intended velocity; the only manipulation concerned the attentional focus direction. Cues were delivered using a standardized script as an initial instruction immediately before each experimental session and were reinforced with a single brief reminder immediately prior to each set. No additional encouragement was provided beyond the scripted statements; to equate researcher contact time, the control group received a neutral statement of comparable duration that contained no attentional focus content. Participants were explicitly instructed not to discuss the cues with others to limit cross-condition contamination. The scripts were: (i) internal focus: “Think about contracting your pectorals and triceps as hard you can”; (ii) external focus: “Maintain your back firmly against the bench throughout the lift and push the bar as fast and hard as possible”; (iii) control: no attentional focus cues, with participants instructed only to complete the maximum possible repetitions (set-to-failure session) and/or to lift with maximal velocity intent (3 × 10 session), without motivational prompts during execution. To further standardize delivery across groups for tone, duration, and number of reminders, reminders were brief and delivered immediately before each set.

### 2.7. Velocity and Lactate Measures

For each participant and set, repetition velocity was operationalized as the concentric barbell velocity (m·s^−1^) provided by the LPT. Several velocity-derived performance metrics were calculated: (i) mean set velocity (Vmean), defined as the arithmetic average of velocities across all successfully completed repetitions within the set; (ii) first-repetition velocity (Vrep1), corresponding to the velocity of repetition 1; (iii) last-repetition velocity (Vlast), defined as the velocity of the final successfully completed repetition in that set; (iv) absolute velocity loss (AbsVL), computed as Vrep1 − Vlast (m·s^−1^); (v) relative velocity loss (VL%), calculated as [(Vrep1 − Vlast)/Vrep1] × 100. Additionally, (vi) the within-set fatigue slope (Vslope) was estimated by fitting a least-squares linear regression of repetition velocity against repetition number (1–10, or up to the last completed repetition when sets were truncated), with more negative coefficients indicating a faster repetition-by-repetition decline. Session-level indices were derived by averaging set-level values across sets 1–3: (viii) mean session velocity, computed as the mean Vmean across sets, and mean session VL% (S1–S3), obtained as the average VL% across sets. Consequently, all velocity-loss metrics were anchored to the first versus last completed repetition, providing a consistent operational definition even when sets were finalized early due to technical failure.

Blood lactate outcomes were derived from five capillary samples obtained during the 3 × 10 protocol. To capture the cumulative metabolic stimulus within the session, lactate area under the curve (LacAUC) was computed across the five time points using the trapezoidal method, yielding a single-session summary of lactate exposure (mmol·L^−1^·arbitrary time units). Session-wide lactate kinetics were examined as repeated measures using Gaussian generalized estimating equations (GEEs) with an exchangeable working correlation structure (lactate ~Group × Time), which accounts for within-participant dependence without requiring estimation of a random-effects variance component. To relate metabolic and mechanical responses at comparable resolution, lactate values were aligned at the set level with velocity-derived VBT metrics calculated from the corresponding set (e.g., Vmean and VL%). Specifically, the post-set lactate concentration for each set (LacPost = LacS1 − LacS3) was introduced as the physiological predictor in set-level GEE coupling models to evaluate whether lactate–velocity associations differed across attentional focus conditions: VL% ~ LacPost × Group + Set × Group (primary coupling model), and Vmean ~ LacPost × Group + Set × Group (secondary model). The LacPost × Group interaction served as the inferential test for group-dependent lactate–velocity coupling.

This coupling analysis, however, should be interpreted with physiological caution. Although lactate sampling was standardized immediately after each set, blood lactate does not represent a purely set-specific metabolic cost. In a repeated-set protocol with 60 s inter-set recovery, each post-set value reflects the combined influence of the immediately preceding set, delayed lactate appearance, ongoing clearance, and cumulative workload from previous sets. Therefore, the group-dependent lactate–VL% association was interpreted as evidence that cueing conditions influenced the time-aligned relationship between mechanical fatigue and systemic metabolic disturbance, rather than as proof that a given set produced a discrete lactate cost per unit of VL%.

### 2.8. Statistical Analysis

All analyses were conducted using Python 3.12 (Python Software Foundation, Beaverton, OR, USA), adopting a two-tailed significance criterion of *α* = 0.05. Assumptions underpinning Gaussian analyses were examined using the Shapiro–Wilk test for normality and Levene’s test for homogeneity of variances when appropriate. Baseline equivalence between groups was evaluated via heteroscedasticity-robust Welch’s one-way ANOVA, with *ω*^2^ reported as the effect size index, and, when applicable, pairwise comparisons were quantified using Hedge’s g with 95% confidence intervals (CIs).

Repetitions to failure during the 60% 1RM protocol were analyzed using a Poisson generalized linear model (GLM) with Group entered as the explanatory factor. The suitability of the Poisson assumption was assessed through dispersion diagnostics (Pearson *χ*^2^/df), with a negative binomial specification considered if overdispersion was detected. Terminal concentric velocity at failure was subsequently compared between groups using Welch’s one-way ANOVA.

During the 3 × 10 protocol, repetition-level concentric velocity (m·s^−1^) was examined using Gaussian linear mixed-effects models (LMMs) incorporating fixed effects for Group × Set × Repetition interactions and a participant-specific random intercept. Repetition number was treated as a continuous, mean-centered covariate to quantify repetition-by-repetition fatigue slopes. Models were estimated using restricted maximum likelihood (REML) for parameter inference and subsequently refitted by maximum likelihood (ML) when conducting likelihood ratio tests (LRT) for comparisons among nested specifications. In parallel, set-derived VBT parameters (e.g., Vmean, VL%, and Vslope) were evaluated with set-level mixed models including Group × Set fixed effects and a random intercept at the participant level. All VL metrics were computed using the first versus last successfully completed repetition within each set (Vrep1 vs. Vlast) to preserve a consistent operational definition when sets were finalized prematurely.

Lactate responses during the 3 × 10 protocol were modeled with Gaussian GEEs using an exchangeable working correlation structure and robust (sandwich) standard errors (lactate ~ Group × Time). GEEs were preferred over a mixed-effects approach because the random-effects variance component approached zero (i.e., singular fit), and also to provide population-averaged estimates that remained robust even if the working correlation was mis-specified. The association between metabolic and mechanical outputs was evaluated via set-level GEE coupling models (three observations per participant) that included lactate-by-group interaction terms: VL% ~ lactate post × Group + Set × Group (primary), and Vmean ~ lactate post × Group + Set × Group (complementary), where lactate post represents the post-set lactate concentrations for sets 1–3 (LacS1–LacS3). Missing data were addressed using available case analyses, without imputation.

To control for multiplicity, Holm adjustment was implemented for a prespecified family of aggregated outcomes, including mean VL% across sets 1–3, mean Vmean across sets 1–3, LacAUC, post-set 3 lactate concentration (LacS3), and post-exercise RPE. Across analyses, Holm-adjusted *p*-values are presented alongside point estimates and 95% CIs.

## 3. Results

Thirty-six participants were randomized to the three cueing conditions. Of these, 34 completed the study; two participants dropped out for injuries unrelated to the study. Baseline characteristics are reported for the randomized/analyzed baseline sample, whereas outcome-specific analyses used the available valid data for each variable. Accordingly, the effective sample size differs across outcomes when valid 3 × 10 velocity data, lactate data, or sufficient within-set repetitions for slope-based estimates were required. The specific denominator used for each analysis is reported in the corresponding table/figure.

Baseline characteristics of the analyzed sample are presented in Table 1. No between-group differences were observed for anthropometric or strength-related variables (all *p* > 0.005). For instance, 1RM values were comparable across groups (Welch’s F = 2.17, *p* = 0.139, *ω*^2^ = 0.070), as were relative strength (Welch’s F = 1.88, *p* = 0.179, *ω*^2^ = 0.042) and body fat percentage (Welch’s F = 1.45, *p* = 0.261, *ω*^2^ = 0.066). See Figure S1 for the participant flow diagram.

### 3.1. Data Completeness, Early Termination, and Operational Definition of Last Completed Repetition

During the 3 × 10 protocol, several sets were finalized early because of technical failure (i.e., two consecutive technique warnings), which led to truncated repetition sets. Because set finalization was triggered by fatigue-related loss of technical execution, truncation should not be interpreted as missing completely at random. Rather, early termination likely reflects the interaction between accumulated fatigue and the participant’s ability to maintain the required technical standard. Accordingly, group- and set-dependent truncation may reduce the precision of late-repetition estimates and should be considered when interpreting derived indices such as VL% and, particularly, Vslope.

Consequently, for all set-level VL outcomes, the last successfully completed repetition was treated as the terminal repetition; thus, Vlast was defined as the velocity of the last completed repetition within-set, and VL% was calculated as [(Vrep1 − Vlast)/Vrep1] × 100. This definition avoids artificial inflation or attenuation of VL that could arise from imputing velocities for repetitions that were not performed. In addition, Vslope was computed only when a sufficient number of repetitions was available, given that slope-based estimates are especially sensitive to reduced repetition trajectories.

**Figure 2:**
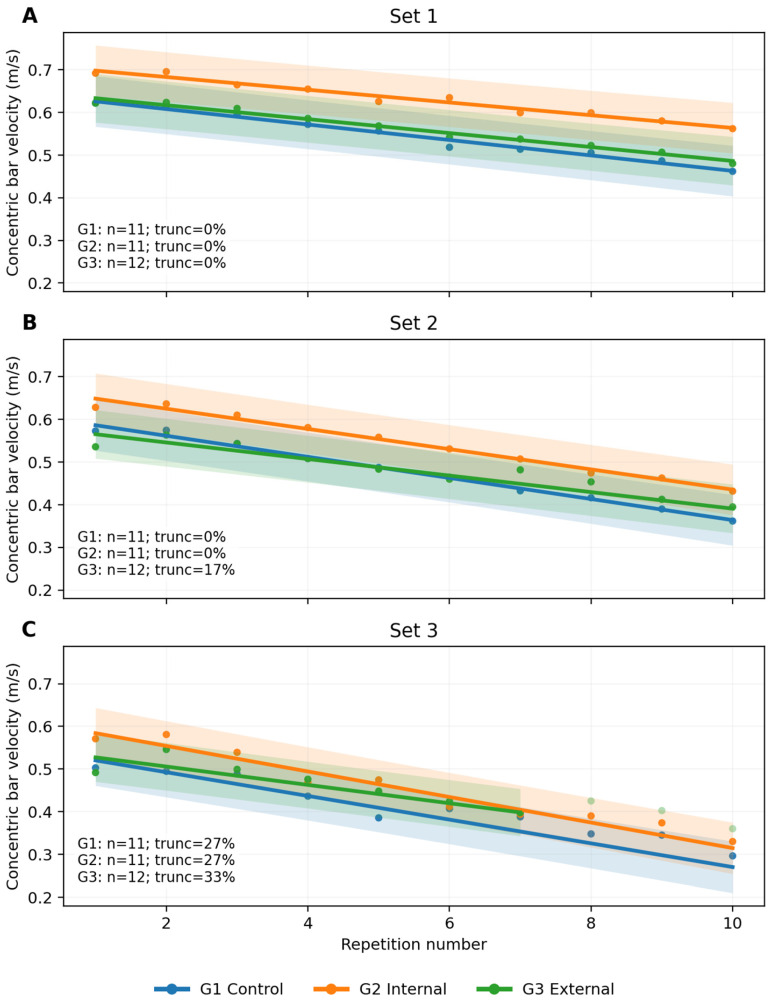
Repetition-by-repetition concentric barbell velocity across sets and attentional focus conditions during the 3 × 10 protocol at 60% 1RM. Note: concentric bar velocity (m·s^−1^) is displayed as a function of the repetition number of set 1–3 (panels **A**–**C**) in the Smith machine BP. Dots represents the observed mean velocity per repetition within each group; solid lines indicate marginal predictions from the repetition-level linear mixed-effects model (LMM; fixed effects); shaded bands depict the 95% CI of the marginal mean. Predicted trajectories are shown only up to the last repetition where the effective sample size per repetition remained ≥70% of the set-specific *n* at repetition 1 (to minimize extrapolation under early set finalization). Inset text reports *n* per set and truncation (%) per group. Trunc: truncation.

**Figure 3:**
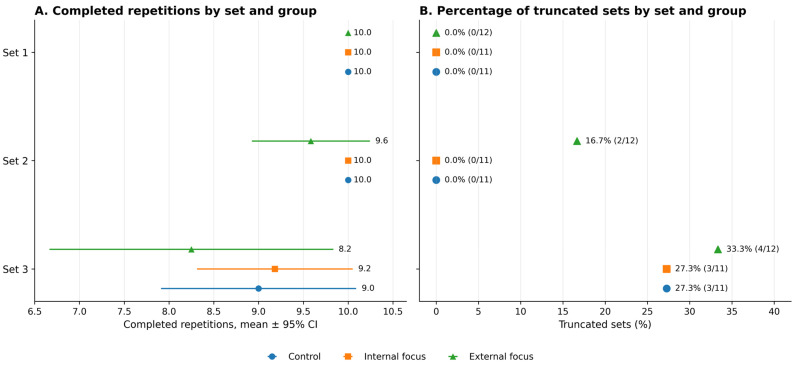
Completed repetitions and truncated sets across the 3 × 10 protocol by cueing condition. Panel (**A**) shows completed repetitions by set and group as mean ± 95% CI. Panel (**B**) shows the percentage of truncated sets by set and group, with the number of truncated sets relative to the number of participants shown in parentheses. A set was considered truncated when fewer than 10 repetitions were completed due to technical failure, defined as two consecutive technique warnings. Inset text reports *n* per set and truncation (%) per group.

The frequency of early finalization increased across successive sets and differed between groups, most notably in set 3, which is pertinent when interpreting late-repetition estimates. Effective sample sizes and truncation percentages by set and condition are reported in Figure 2 and summarized in Table 2, including effective n per cell outcomes requiring complete within-set trajectories (e.g., Vslope). Furthermore, to provide additional context for interpreting VL% and lactate responses, achieved volume and truncation patterns are presented in Figure 3. The data regarding late-set mechanical and metabolic outcomes should be interpreted in light of the achieved volume and fatigue-related occurrence of technical failure.

### 3.2. Experimental Session: Set-to-Failure at 60% 1RM

Repetition to failure during the 60% 1RM protocol was modeled with a Poisson GLM including Group as the explanatory factor. Dispersion diagnostics supported the Poisson assumption (Pearson χ^2^/df = 1.03), and no compelling evidence emerged for between-group differences in repetitions to failure.

Terminal concentric velocity at failure was evaluated across groups using Welch’s ANOVA and likewise did not indicate clear group effects (F = 1.175, *p* = 0.328, *ω*^2^ = 0.045). Means terminal velocities were 0.210 m·s^−1^ for the internal focus condition, 0.169 m·s^−1^ for the external focus condition, and 0.167 m·s^−1^ for the control condition.

### 3.3. Experimental Session: Repetition-Level Velocity Dynamics During the 3 × 10 Protocol

Repetition-level concentric velocity was modeled using a LMM including fixed effects for the Group × Set × Repetition interaction and a participant-specific random intercept. Across the protocol, a clear fatigue-related decline in velocity was evident.

Set effect (accumulated fatigue): velocity decreased progressively from set to set (overall set effect *p* < 0.001). Compared with set 1, lower velocities were observed in set 2 and set 3; at the mid-set reference point, the estimated reductions were approximately −0.069 m·s^−1^ (set 2 vs. set 1) and −0.149 m·s^−1^ (set 3 vs. set 1).

Repetition effect (within-set fatigue): within each set, velocity declined across repetitions (*p* < 0.001), with an average decrease of approximately −0.018 m·s^−1^ per repetition in the reference condition. This decline was amplified in later sets, as indicated by a significant Set × Repetition interaction.

Group contribution: inclusion of group-related terms significantly improved model fit (likelihood ratio test, LR *χ*^2^(12) = 42.26, *p* < 0.001). Incorporating group-by-repetition interactions—reflecting between-group differences in fatigue pattern—yielded further improvement (LR *χ*^2^(6) = 14.90, *p* = 0.021). Visual inspection suggested that the internal focus group tended to exhibit higher velocities in the early phase of the protocol, whereas between-group separation attenuated as accumulated fatigue increased (Figure 2).

### 3.4. Set-Level VBT Outcomes Derived from Repetition Data

A set-level mixed-effects model was applied to the derived VBT outcomes, including Group × Set and a participant-specific random intercept. Descriptive values are provided in Table 2.

Mean set velocity (Vmean): Vmean decreased across successive sets (set effect: *p* < 0.001). In set 1, the internal focus group exhibited a higher Vmean than the control group by approximately +0.086 m·s^−1^ (*p* = 0.032). Evidence for Group × Set interactions was inconclusive, aligning with the observed convergence of velocity profiles in the later sets as accumulated fatigue increased.

Relative velocity loss (VL%). VL% rose substantially across sets, with set 3 showing an increase of roughly +36.6% relative to set 1 (*p* < 0.001). In the set-level summary model, the main effect of group on VL% did not reach statistical significance, suggesting that mean VL% did not differ consistently across instructional conditions when evaluated at the aggregated set level.

### 3.5. Internal Load: Lactate Kinetics During the 3 × 10 Protocol

Lactate kinetics were evaluated using Gaussian GEEs with an exchangeable working correlation structure and robust (sandwich) standard errors. Lactate rose markedly over time (Time: *χ*^2^(4) = 244.56, *p* < 0.001). Although the overall main effect of group was not significant (Group: *χ*^2^(2) = 2.07, *p* = 0.355), a significant Group × Time interaction was observed (*χ*^2^(8) = 26.22, *p* < 0.001), indicating that lactate time–course patterns differed between conditions. Specifically, the external focus condition exhibited higher lactate concentrations during the early phase (post-set 1 and post-set 2), whereas values converged across groups by post-set 3 and 30 s after the final set.

**Figure 4:**
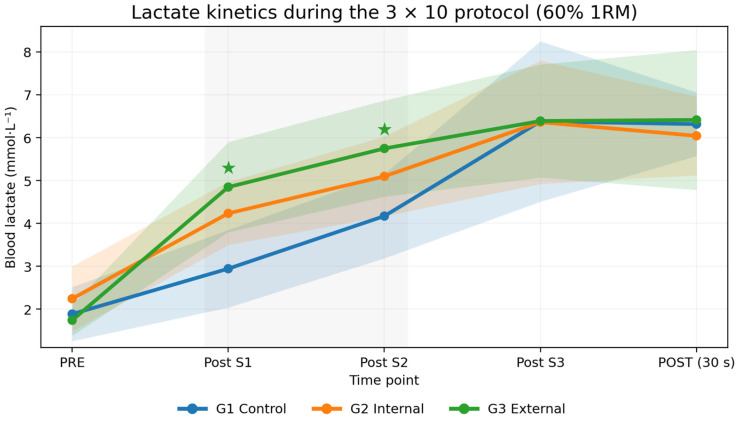
Blood lactate kinetics across the 3 × 10 protocol in Smith machine BP (60% 1RM) by attentional focus condition. Note: lactate concentration (mmol·L^−1^) at five repeated time points (pre, post-set 1, 2, and 3, and 30 s post-set 3) is plotted for each group. Lines represent group means and shaded bands indicate 95% CIs. Green stars highlight time points contributing to the significant Group × Time interaction from the Gaussian GEE model (exchangeable working correlation; robust standard errors). Shaded areas denote 95% confidence intervals.

Descriptively, lactate remained elevated in the external focus group at the first two post-set measurements, with between-group differences diminishing at later time points (post-set 3 and 30 s after the final set). Group means across all five time points and LacAUC are reported in Table 3 and visualized in Figure 4.

### 3.6. Group-Dependent Lactate–Mechanical Fatigue Coupling

To evaluate whether attentional focus modulated the coupling between metabolic stress and mechanical fatigue, set-level GEE coupling models were estimated using three observations per participant (sets 1–3), pairing each post-set lactate value with the VBT metrics derived from the corresponding set.

**Figure 5:**
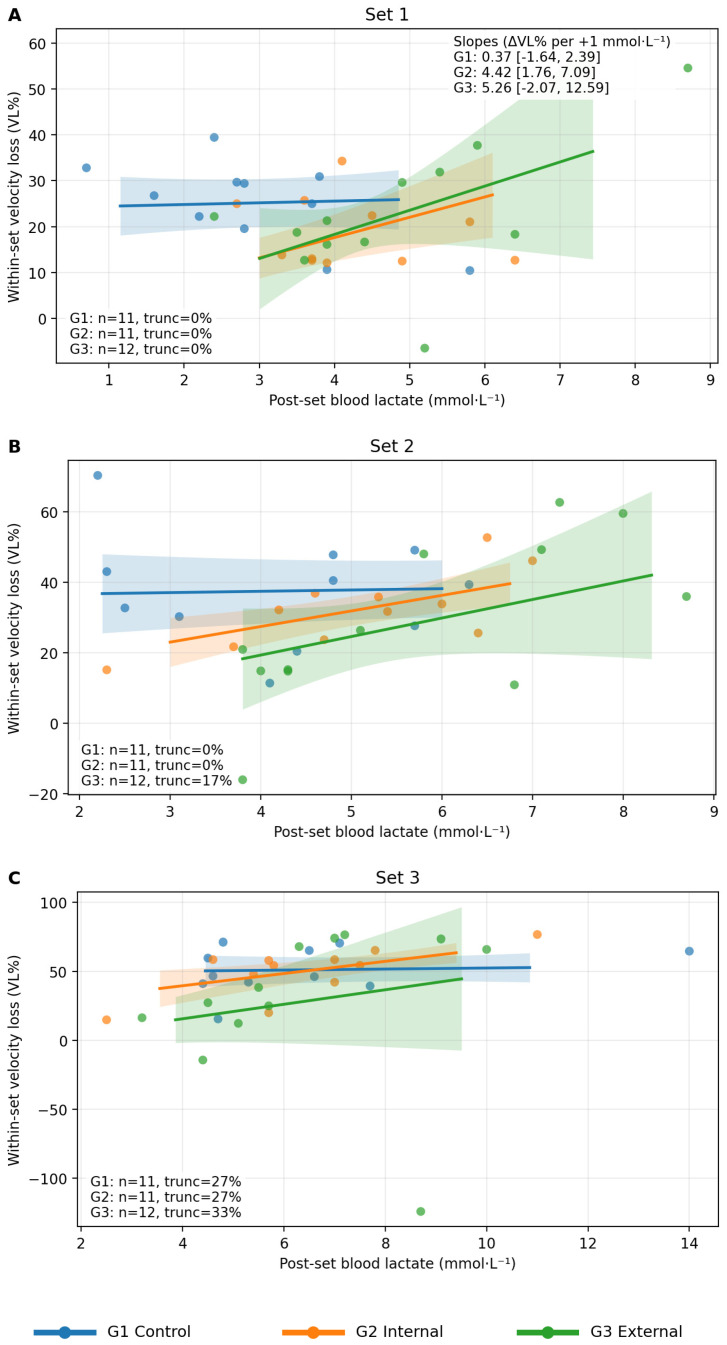
Group-dependent association between post-set blood lactate and within-set VL across different groups. Note: set-level mechanical–metabolic coupling during submaximal BP performed with maximal intended concentric velocity. Scatterplots depict VL% versus post-set blood lactate for set 1 (**A**), set 2 (**B**), and set 3 (**C**) across groups (G1 control, G2 internal focus, G3 external focus). Regression lines and 95% CI bands derive from the final Gaussian GEE model with exchangeable correlation and sandwich SE, ensuring consistency between visual inference and primary statistical testing. Truncation reflects early set termination due to technical failure; inset text reports *n* per set and truncation (%) per group; VL% is operationally defined using the first versus last completed repetition. Shaded areas denote 95% confidence intervals.

Primary coupling model (VL% as outcome). In the primary model (VL% ~ lactate post × Group + Set × Group), the main effect of post-set lactate was not significant (*p* = 0.968). In contrast, the lactate Post-Set × Group interaction was highly significant *χ*^2^(2) = 80.42, *p* < 0.001), demonstrating that the lactate–VL% association was strongly dependent on instructional condition. Estimated slopes (ΔVL% per +1 mmol·L^−1^ lactate; 95% CI) were: (i) internal focus: 5.27 (1.51, 9.03); (ii) external focus: 13.60 (11.46, 15.75); (iii) control: 0.04 (−2.01, 2.10). Thus, the external focus group exhibited a substantially stronger positive relationship between post-set lactate and within-set relative VL (Figure 5).

Complementary coupling model (Vmean as outcome). In the complementary model (Vmean ~ lactate post × Group + Set × Group), the lactate–velocity coupling was most apparent under external focus, where higher post-set lactate was associated with lower mean set velocity (slope: −0.0204 m·s^−1^ per +1 mmol·L^−1^; 95% CI: −0.0290, −0.0117). Corresponding estimates for the control and internal focus conditions were small and not statistically significant. The full GEE outputs for both models are presented in Table 4.

### 3.7. Multiplicity-Controlled Aggregated Outcomes

To yield a conservative summary consistent with the prespecified analysis plan, multiplicity was addressed using the Holm procedure applied to a predefined family of aggregated outcomes: mean VL% across sets 1–3, mean Vmean across sets 1–3, LacAUC, post-set 3 lactate, and post-exercise RPE. Following Holm adjustment, only RPE remained statistically significant (*p* = 0.004; *ω*^2^ = 0.276). Pairwise contrasts indicated greater perceived exertion in the external focus group relative to both the control group and the internal focus condition. Because Hedge’s *g* was calculated as the comparator group minus the external focus group, the negative values indicate a higher RPE in the external focus condition (control vs. external Hedge’s *g* = −1.50, 95% CI −2.43 to −0.56) and internal focus condition (internal vs. external Hedge’s *g* = −1.37, 95% CI −2.24 to −0.49). In contrast, the other aggregated outcomes did not exhibit adjusted between-group differences (all *p* Holm = 1.000), implying that instructional effects are more evident in time-dependent patterns and lactate–velocity coupling than in session-averaged mechanical or lactate summaries.

## 4. Discussion

### 4.1. Mechanical Fatigue Responses Under Standardized Cueing Conditions

The present study aimed to determine whether different attentional focus instructions (internal, external, and control) could modulate velocity-based training (VBT) profiles during the Smith machine BP. Beyond the mechanical outputs, we also examined how these cues influenced metabolic stress—specifically lactate kinetics—and the overall development of fatigue throughout the set. Our findings provide critical insights into how the standardization of verbal cues affects the interpretation of VL as a primary training criterion. The main results show that the dominant mechanical response in this study was a pronounced reduction in barbell velocity as repetitions accumulated within each set and as successive sets unfolded across the session, aligning with the established view of movement velocity as a highly accurate indicator of neuromuscular fatigue during RT [21]. From an applied VBT perspective, these data indicate that, even when relative intensity is fixed (60% 1RM) and concentric actions are performed with standardized maximal intent, the velocity–time trajectory reflects both within-set fatigue (repetition-to-repetition decay) and between-set accumulated fatigue. This supports the ecological validity of derived indices such as Vmean and VL% as practical descriptors of the mechanical expression of effort [5]. Extending this general fatigue signature, between-group summaries suggested that Vmean was modestly higher in the internal focus group than in the control during the first set, whereas VL% rose substantially from early to late sets across all conditions. Collectively, this pattern is consistent with the premise that attentional focus can modulate movement organization and performance expression without necessarily yielding large shifts in aggregated fatigue indices when key constraints (e.g., load, pacing, recovery) restrict the available solution space [7,9].

At this point, a brief methodological clarification is warranted to ensure that the interpretation of VL% remains explicit and defensible. In the present study, VL% was calculated using the first repetition versus the last repetition actually performed within each set (i.e., “last completed repetition”), an operational choice that is methodologically appropriate when sets are truncated due to technical failure or predefined stopping criteria [14]. This procedure avoids imputing velocities for repetitions that were not executed and maintains VL% as a valid indicator of the fatigue state attained at the point of set finalization, consistent with common VBT conventions that quantify within-set VL using the slowest/last completed repetition [21,22]. Nevertheless, this definition entails that effective sample sizes may differ across sets and outcomes—particularly for slope-based indices such as Vslope when fewer repetitions are available—such that null between-group differences in averaged VL% should be interpreted in conjunction with repetition-level modeling and the observed lactate–VBT coupling, rather than taken as conclusive evidence that attentional focus lacks mechanical relevance [5].

### 4.2. Lactate Kinetics and Lactate–VL% Coupling

In concert with the mechanical findings, blood lactate rose markedly across the protocol, as expected during a moderate-load, high-repetition BP task, indicating an increasing glycolytic contribution and a progressive systemic metabolic disturbance elicited by repeated sets interspersed with short recovery intervals [23]. Beyond this pronounced temporal effect, the observed Group × Time interaction implies that attentional focus cues were associated with distinct lactate trajectories, most notably during the initial phase of the session, whereas concentrations subsequently converged toward a comparable late-session plateau following the third set and the early recovery period. This qualitative profile accords with the motor control literature suggesting that attentional focus can influence movement economy and the energetic cost of performance, particularly when accumulating fatigue progressively constrains coordination and force production strategies [8,10]. Within a RT context, verbal cueing has been linked to shifts in neuromuscular activation patterns and perceptual responses despite identical external loading, indicating that internal load proxies—including lactate and RPE—may be especially responsive to how instructions are framed [11,24]. The lactate sampling strategy should be interpreted within its intended temporal scope. The post-set samples were standardized to capture early systemic metabolic disturbance during the repeated-set protocol, rather than peak lactate concentration or full post-exercise lactate clearance. Because blood lactate kinetics may continue to evolve beyond the 30 s post-exercise measurement, LacAUC should be understood as a standardized within-protocol summary of early lactate exposure across the five sampling points, expressed in arbitrary time units, rather than as a complete representation of total lactate accumulation or recovery kinetics.

Importantly, the most distinctive finding of the present study extends beyond a group-dependent lactate time course to the observation that the link between post-set lactate and within-set mechanical fatigue (VL%) was not consistent across conditions, but rather appeared to be moderated by attentional focus. Conceptually, this suggests that, for a given set, the degree to which metabolic disturbance covaries with the mechanical expression of fatigue may be contingent on the instructional constraint imposed on the performer. This interpretation is compatible with theoretical frameworks positing that instructional cues can alter the control processes governing movement execution, where an external focus may promote more automatic regulation and different coordination solutions, whereas an internal focus may elicit more consciously controlled strategies [9,12]. The present findings should not be interpreted as evidence that VL inherently has a different physiological meaning across cueing conditions. A more defensible interpretation is that, under the present 60% 1RM Smith machine BP protocol, assigned cueing conditions modified the observed statistical association between post-set lactate and VL%. From an applied standpoint, this suggests that the relationship between VL% and systemic internal load markers may vary according to the cueing condition. However, this should not be interpreted as a discrete physiological cost per unit of velocity decline, because post-set blood lactate reflects delayed kinetics, ongoing clearance, and cumulative workload across the repeated-set protocol. This interpretation is consistent with evidence that verbal instructions can influence neuromuscular strategies during BP under identical external loads [11], and with broader arguments that mechanical and internal load markers may become partially decoupled when task constraints or execution strategies differ [25,26].

Notably, this interaction-based finding should be distinguished from the multiplicity-controlled aggregated outcomes. After Holm correction across the prespecified family of primary aggregated outcomes, only post-session RPE remained statistically significant. Therefore, the lactate–VL% moderation should be interpreted as an important but model-dependent finding within this protocol, rather than as evidence of a consistent between-group effect across all principal mechanical and physiological outcomes. Replication in larger samples and alternative experimental designs is needed before this association can be considered robust for broader VBT prescription.

### 4.3. Methodological Implications for VBT Research and Applied Monitoring

These findings become particularly informative when interpreted through the contemporary VBT lens. In applied VBT, VL is commonly used as a pragmatic proxy for mechanical fatigue and as an operational rule for dosing effort and volume within a set [5]. Consequently, the central methodological implication of the observed moderation is that, if attentional focus shifts the lactate–VL% coupling, then identical VL% values may not correspond to equivalent internal load profiles across different cueing conditions, even when relative intensity is standardized. This point is critical because VL-driven prescriptions implicitly assume that attaining a given VL threshold (e.g., 10–30% or higher) produces comparable fatigue exposure and training dose across sessions and individuals [4,5].

Moreover, the practical utility of VL is closely tied to its association with proximity to failure and the effective level of exertion, thereby operationalizing constructs such as RIR in day-to-day monitoring [21]. If attentional focus alters how metabolic strain co-develops with VL, two sets terminating at the same VL% could still differ in the underlying internal load (and potentially perceived cost), undermining dose comparability when verbal cues are not explicitly standardized. This concern is amplified by longitudinal BP interventions demonstrating that different VL thresholds elicit distinct training exposures and adaptations despite similar relative intensities, emphasizing that VL functions not only as a descriptive marker but also as a programming variable with tangible consequences [22]. Therefore, beyond suggesting that attentional focus may influence mean velocity and fatigue dynamics at specific stages, the present data more fundamentally indicate that instructional framing can modify the physiological interpretation of a given VL%. This provides a concrete rationale for explicitly standardizing and reporting attentional cues in VBT research and applied monitoring contexts, particularly when VL-derived indices are interpreted alongside physiological markers such as blood lactate to infer internal load [5,23]. Accordingly, future VBT studies should report cueing scripts verbatim and treat instruction as a key control variable when comparing VL-based dosing across conditions.

From an applied perspective, these findings indicate that, during moderate-load Smith machine BP performed with maximal concentric intent, attentional cueing should be considered a meaningful methodological determinant rather than a procedural detail. First, when VL% is employed either as a stopping rule or as a descriptive indicator of fatigue, the cueing approach should be explicitly reported and kept consistent across sessions to preserve the interpretability of both mechanical and physiological readouts [5]. Second, when blood lactate (or alternative internal load proxies) is collected to contextualize VBT-derived indices, the observed moderation pattern suggests that cueing may systematically shape lactate–fatigue coupling; therefore, interaction-based modeling frameworks (e.g., mixed or marginal models testing lactate × group terms) are preferable to simple pooled correlations that assume homogeneity of relationships across conditions [26]. Finally, given evidence that attentional focus can modify BP execution strategies [11] and that instructional effects may depend on task constraints, the Smith machine offers a controlled platform to isolate cue-driven effects, but it also necessitates caution when extrapolating to free-weight BP, where stabilization demands and movement degrees of freedom are greater [27,28,29].

### 4.4. Limitations and Future Directions

Several limitations should be considered when interpreting these findings:

(i) The parallel between-group design, with approximately 11 participants per condition, constrains statistical precision for higher-order interaction terms (e.g., Group × Set × Repetition), which are typically small-to-moderate in attentional focus research.
(ii) Premature set finalization also reduced the number of repetitions available for slope-based estimates and increased informational imbalance across cells; although defining VL% using the “last completed repetition” is methodologically sound, it likely increases uncertainty for outcomes such as Vslope. The achieved volume and truncation data should also be considered when interpreting the VL% and lactate findings. Because truncated sets reflected fatigue-related inability to maintain the required technical standard, group- and set-specific differences in completed repetitions may have contributed to the observed mechanical and metabolic patterns. Accordingly, the lactate–VL% coupling results should be interpreted as time-aligned associations within the achieved repeated-set workload, rather than as estimates obtained under perfectly equivalent volume completion across all participants and cueing conditions.
(iii) An additional limitation is that lactate was only monitored up to 30 s after the final set. Therefore, the present data cannot determine whether between-condition differences would persist, increase, or disappear during later recovery. Future studies should include additional post-exercise sampling points to characterize peak lactate responses and subsequent clearance kinetics more completely.
(iv) Concerning post-set blood lactate provides a systemic and temporally delayed marker of metabolic disturbance. Thus, the present coupling model cannot isolate the metabolic cost of each individual set, particularly under short inter-set recovery. Future studies could combine more frequent blood sampling, longer recovery intervals, near-infrared spectroscopy, electromyography, or other local physiological measures to better characterize the temporal and mechanistic relationship between mechanical fatigue and metabolic stress.
(v) A limitation of the present study is the absence of a formal manipulation check to verify adherence to the assigned attentional focus during each set. Although cue delivery was standardized, researcher-controlled, reinforced before each set, and matched for contact time across groups, participants’ actual attentional state was not directly assessed. Accordingly, the observed differences should be interpreted as effects of assigned cueing conditions, rather than definitive evidence that an internal or external focus was consistently maintained throughout the task. Futures studies should include manipulation checks, such as immediate post-set self-reports or validated attentional focus adherence scales, to determine whether the prescribed focus was adopted and sustained during fatiguing resistance exercise.
(vi) Cue content differed across conditions despite matching cue duration, delivery, and frequency. Although the corrected external cue aimed to standardize body position while directing attention toward rapid bar displacement, it included more movement- and technique-related information than the control condition. Therefore, the observed effects cannot be attributed solely to attentional direction, as cue content may also have influenced execution strategy, stabilization demands, or perceived effort.
(vii) Although technical failure was defined a priori and monitored by two investigators, no formal inter-rater reliability statistic was calculated for warning decisions. Because these decisions directly affected repetitions to failure, truncation, Vlast, VL%, and Vslope, future studies should implement inter-rater standardization procedures or video-based reliability checks for technical failure classification.
(viii) In addition, the results were obtained using the Smith machine BP and may not generalize directly to free-weight conditions, where stabilization demands and movement degrees of freedom are greater.

## 5. Conclusions

In summary, the mechanical signature of fatigue, characterized by repetition- and set-driven velocity decrements, behaved as expected under a standardized 60% 1RM Smith machine BP protocol. The hypotheses were partially supported, as the assigned cueing conditions did not consistently alter mean VL% at the set or session level, but were associated with differences in early lactate kinetics and, most notably, in the observed association between post-set lactate and VL%. These findings suggest that standardized cueing conditions may influence the observed mechanical–metabolic coupling between post-set lactate and VL% in this protocol, rather than proving that VL inherently has a different physiological meaning. However, because the strongest evidence emerged from interaction-based modeling rather than from a consistent pattern across multiplicity-controlled aggregated outcomes, this interpretation should be considered provisional and requires replication. This pattern carries direct methodological implications for VBT research and applied monitoring, supporting the need to report cueing scripts verbatim and to standardize verbal instructions, because such cues may influence the relationship between VL-derived mechanical fatigue indices and internal load markers. When VL% is considered alongside internal load markers such as lactate or RPE, the cueing context should be treated as a relevant methodological factor rather than a trivial procedural detail.

## Figures and Tables

**Table 1:** Baseline characteristics of participants by attentional focus group.

Variable	Control	Internal	External	Welch F	*p*-Value	*ω* ^2^
Age (years)	28.09 (23.04, 33.14) ± 7.52	26.45 (22.97, 29.94) ± 5.18	25.08 (20.37, 29.80) ± 7.42	0.45 (2, 20.12)	0.643	0.000
Body mass (kg)	85.89 (72.50, 99.28) ± 19.93	74.89 (69.54, 80.24) ± 7.96	75.71 (68.20, 83.22) ± 11.82	1.41 (2, 18.79)	0.269	0.061
Height (cm)	177.64 (172.81, 182.46) ± 7.19	175.55 (172.48, 178.61) ± 4.57	177.25 (173.17, 181.33) ± 6.43	0.44 (2, 19.95)	0.648	0.000
Body fat (%)	21.03 (14.61, 27.44) ± 9.55	16.75 (13.81, 19.68) ± 4.36	15.86 (13.87, 17.85) ± 3.13	1.45 (2, 17.93	0.261	0.066
1RM load (kg)	113.64 (94.88, 132.39) ± 27.91	92.12 (79.05, 105.18) ± 21.62	102.50 (87.72, 117.28) ± 23.26	2.17 (2, 21.10)	0.139	0.070
Velocity at 1RM (m·s^−1^)	0.159 (0.132, 0.186) ± 0.040	0.178 (0.152, 0.203) ± 0.042	0.141 (0.103, 0.178) ± 0.059	1.64 (2, 21.39)	0.218	0.045
Relative strength (kg·kg^−1^)	1.34 (1.14, 1.55) ± 0.30	1.18 (1.02, 1.34) ± 0.24	1.35 (1.23, 1.48) ± 0.20	1.88 (2, 19.64)	0.179	0.042
60% 1RM load (kg)	68.18 (56.93, 79.43) ± 16.75	55.27 (47.43, 63.11) ± 12.97	61.50 (52.63, 70.37) ± 13.95	2.17 (2, 21.10)	0.139	0.070

Note: values are mean (95% CI) ± SD. Groups: control *n* = 11; internal *n* = 12; external *n* = 13. Between-group comparisons used Welch’s ANOVA (unequal variances), reporting Welch F, *p*-value, and omega squared (*ω*^2^). 1RM: one-repetition maximum.

**Table 2:** Bar velocity fatigue metrics by set and group.

Set	Variable	Control (*n* = 11)	Internal (*n* = 11)	External (*n* = 12)
Set 1	Vmean (m·s^−1^)	0.544 (0.492, 0.597) ± 0.079	0.631 (0.573, 0.688) ± 0.085	0.560 (0.511, 0.608) ± 0.076
	Vrep1 (m·s^−1^)	0.622 (0.562, 0.682) ± 0.089	0.692 (0.625, 0.758) ± 0.099	0.622 (0.575, 0.669) ± 0.074
	Vlast (m·s^−1^)	0.462 (0.417, 0.507) ± 0.067	0.562 (0.502, 0.621) ± 0.088	0.480 (0.415, 0.545) ± 0.103
	VL (%)	25.17 (19.15, 31.20) ± 8.97	31.18 (12.27, 50.09) ± 31.30	22.79 (13.33, 32.24) ± 14.89
	Vslope (m·s^−1^·rep^−1^)	−0.0181 (−0.0227, −0.0135) ± 0.0069	−0.0149 (−0.0179, −0.0120) ± 0.0044	−0.0163 (−0.0215, −0.0112) ± 0.0081
Set 2	Vmean (m·s^−1^)	0.475 (0.405, 0.545) ± 0.104	0.542 (0.481, 0.604) ± 0.092	0.481 (0.418, 0.544) ± 0.099
	Vrep1 (m·s^−1^)	0.573 (0.500, 0.645) ± 0.108	0.628 (0.566, 0.690) ± 0.092	0.536 (0.463, 0.609) ± 0.115
	Vlast (m·s^−1^)	0.362 (0.274, 0.449) ± 0.130	0.432 (0.348, 0.515) ± 0.124	0.372 (0.296, 0.447) ± 0.119
	VL (%)	37.55 (26.91, 48.18) ± 15.84	32.35 (25.08, 39.61) ± 10.81	37.28 (14.47, 60.10) ± 35.91
	Vslope (m·s^−1^·rep^−1^)	−0.0247 (−0.0312, −0.0182) ± 0.0096	−0.0237 (−0.0274, −0.0200) ± 0.0055	−0.0192 (−0.0287, −0.0097) ± 0.0141
Set 3	Vmean (m·s^−1^)	0.401 (0.314, 0.488) ± 0.130	0.457 (0.380, 0.534) ± 0.115	0.444 (0.380, 0.508) ± 0.101
	Vrep1 (m·s^−1^)	0.503 (0.410, 0.595) ± 0.138	0.571 (0.500, 0.642) ± 0.105	0.492 (0.424, 0.559) ± 0.107
	Vlast (m·s^−1^)	0.252 (0.158, 0.345) ± 0.139	0.298 (0.191, 0.406) ± 0.160	0.326 (0.206, 0.445) ± 0.188
	VL (%)	61.75 (42.56. 80.94) ± 28.57	61.04 (41.09, 80.98) ± 29.69	54.10 (27.85, 80.34) ± 41.31
	Vslope (m·s^−1^·rep^−1^)	−0.0309 (−0.0403, −0.0215) ± 0.0122	−0.0305 (−0.0388, −0.0222) ± 0.0107	−0.0216 (−0.0363, −0.0069) ± 0.0176

Note: Vmean is the mean concentric velocity across repetitions within each set. Vrep1 is repetition 1 velocity. Vlast is the velocity of the last completed repetition. VL% corresponds to within-set relative VL computed as [(Vrep1 − Vlast)/Vrep1)] × 100. Vslope is the intra-set fatigue slope from an OLS regression of velocity on repetition number (Vslope was computed only when ≥8 repetitions were available). Effective *n* (Vslope): Set 1, control = 11, internal = 11, external = 12; Set 2, control = 11, internal = 11, external = 11; Set 3, control = 9, internal = 9, external = 8. Completion of 10 repetitions/*n* with set data: Set 1, control 11/11, internal 11/11, external 12/12; Set 2, control 11/11, internal 11/11, external 10/12; Set 3, control 8/11, internal 8/11, external 8/12.

**Table 3:** Internal load summary indices and VBT dose metrics.

Variable	Control (*n* = 11)	Internal (*n* = 11)	External (*n* = 12)	Welch *p*	*ω* ^2^	Holm *p*
Lactate Pre-Set 1 (mmol·L^−1^)	1.89 (1.26, 2.52) ± 0.88	2.25 (1.49, 3.00) ± 1.13	1.74 (1.38, 2.10) ± 0.57	–––	–––	–––
Lactate Post-Set 1 (mmol·L^−1^)	2.95 (2.04, 3.85) ± 1.35	4.24 (3.50, 4.97) ± 1.09	4.85 (3.80, 5.90) ± 1.66	–––	–––	–––
Lactate Post-Set 2 (mmol·L^−1^)	4.17 (3.19, 5.16) ± 1.46	5.10 (4.17, 6.03) ± 1.39	5.75 (4.63, 6.87) ± 1.77	–––	–––	–––
Lactate Post-Set 3 (mmol·L^−1^)	6.38 (4.51, 8.25) ± 2.78	6.36 (4.93, 7.80) ± 2.14	6.39 (5.07, 7.71) ± 2.08	0.999	0.000	1.000
Lactate 30 s Post-Set 3 (mmol·L^−1^)	6.32 (5.58, 7.06) ± 1.10	6.05 (5.13, 6.97) ± 1.37	6.42 (4.78, 8.05) ± 2.57	–––	–––	–––
LacAUC (mmol·L^−1^·a.u.)	17.39 (13.99, 20.80) ± 4.76	19.85 (16.67, 23.02) ± 4.72	21.07 (16.93, 25.21) ± 6.51	0.301	0.016	1.000
Vmean (Session 1–3) (m·s^−1^)	0.474 (0.406, 0.541) ± 0.101	0.543 (0.480, 0.607) ± 0.095	0.495 (0.442, 0.547) ± 0.083	0.252	0.036	1.000
VL% (Session 1–3)	41.49 (30.67, 52.30) ± 16.10	46.99 (31.01, 62.97) ± 26.44	38.05 (19.97, 56.14) ± 28.47	0.715	0.000	1.000
Post-Session RPE	8.32 (8.09, 8.54) ± 0.34	8.08 (7.67, 8.48) ± 0.67	8.83 (8.63, 9.04) ± 0.33	<0.001	0.276	0.004

Note: blood lactate was sampled at baseline (Pre-Set 1), immediately after sets 1–3, and 30 s after set 3. LacAUC denotes trapezoidal area under the lactate curve across the five time points (arbitrary time units). For prespecified primary outcomes, Welch *p*, *ω*^2^, and the Holm-adjusted *p* for all variables are provided.

**Table 4:** Generalized estimating equation outputs for the primary and secondary lactate–velocity coupling models.

Model	Outcome	Predictor	β	Robust SE	95% CI	*p*-Value
Primary	VL%	Intercept	25.04	4.14	16.93 to 33.16	<0.001
		Internal focus	−28.69	7.40	−43.20 to −14.17	<0.001
		External focus	−68.23	8.10	−84.11 to −52.34	<0.001
		Set 2	12.32	4.41	3.67 to 20.96	0.005
		Set 3	36.43	10.25	16.32 to 56.53	<0.001
		Set 2 × Internal focus	−3.19	5.94	−14.84 to 8.45	0.591
		Set 3 × Internal focus	−5.26	15.08	−34.82 to 24.29	0.727
		Set 2 × External focus	−10.06	9.29	−28.28 to 8.15	0.279
		Set 3 × External focus	−26.09	13.20	−51.96 to −0.21	0.048
		Post-set lactate	0.04	1.04	−2.01 to 2.09	0.968
		Post-set lactate × Internal focus	5.22	2.18	0.94 to 9.50	0.017
		Post-set lactate × External focus	13.56	1.51	10.58 to 16.53	<0.001
Secondary	Vmean	Intercept	0.55	0.03	0.48 to 0.62	<0.001
		Internal focus	0.11	0.05	0.003 to 0.21	0.045
		External focus	0.10	0.04	0.01 to 0.19	0.023
		Set 2	−0.06	0.01	−0.09 to −0.03	<0.001
		Set 3	−0.13	0.03	−0.19 to −0.06	<0.001
		Set 2 × Internal focus	−0.01	0.01	−0.05 to 0.01	0.361
		Set 3 × Internal focus	−0.02	0.04	−0.11 to 0.06	0.571
		Set 2 × External focus	0.005	0.02	−0.03 to 0.04	0.823
		Set 3 × External focus	0.04	0.03	−0.03 to 0.12	0.234
		Post-set lactate	−0.004	0.006	−0.01 to 0.009	0.572
		Post-set lactate × Internal focus	−0.005	0.010	−0.02 to 0.01	0.626
		Post-set lactate × External focus	−0.017	0.008	−0.03 to −0.002	0.029

Note: values are regression coefficients from Gaussian generalized estimating equation models with an exchangeable working correlation structure and robust standard errors. The primary model used VL% as the outcome; the secondary model used Vmean as the outcome. Control and Set 1 were used as reference categories. The models included post-set lactate, group, set, post-set lactate × group, and set × group terms. VL%, velocity loss percentage; Vmean, mean concentric velocity; CI, confidence interval.

## Data Availability

Data are available from the corresponding author upon reasonable request. The dataset was also uploaded to the journal submission platform during the manuscript submission process.

## Supplementary Materials

The following supporting information can be downloaded at: https://www.mdpi.com/article/10.3390/jfmk11020189/s1, Figure S1: Participant flowchart.

## Abbreviations

The following abbreviations are used in this manuscript:

VL	Velocity loss
VBT	Velocity-based training
BP	Bench press
1RM	One-repetition maximum
GEEs	Generalized estimating equations
RT	Resistance training
VL%	Velocity loss percentage
LPT	Linear position transducer
RPE	Rate of perceived exertion
LacAUC	Lactate area under the curve
Vmax	Maximum concentric velocity
Vmean	Mean set velocity
Vrep1	First-repetition velocity
Vlast	Last-repetition velocity
AbsVL	Absolute velocity loss
Vslope	Within-set fatigue slope
LacPost	Post-set lactate
ANOVA	Analysis of variance
CI	Confidence interval
GLM	Generalized linear model
LMM	Linear mixed-effects model
REML	Restricted maximum likelihood
ML	Maximum likelihood
LRT	Likelihood ratio test
LacS1	Post-set one lactate
LacS3	Post-set three lactate
