# Supplementary figures and images for: Attentional Cueing Modifies the Observed Association Between Post-Set Lactate and Velocity Loss During Smith Machine Bench Press
Source: J Funct Morphol Kinesiol. 2026 May 11;11(2):189. doi: 10.3390/jfmk11020189 (PMC13214768; doi:10.3390/jfmk11020189)

## Supplementary file

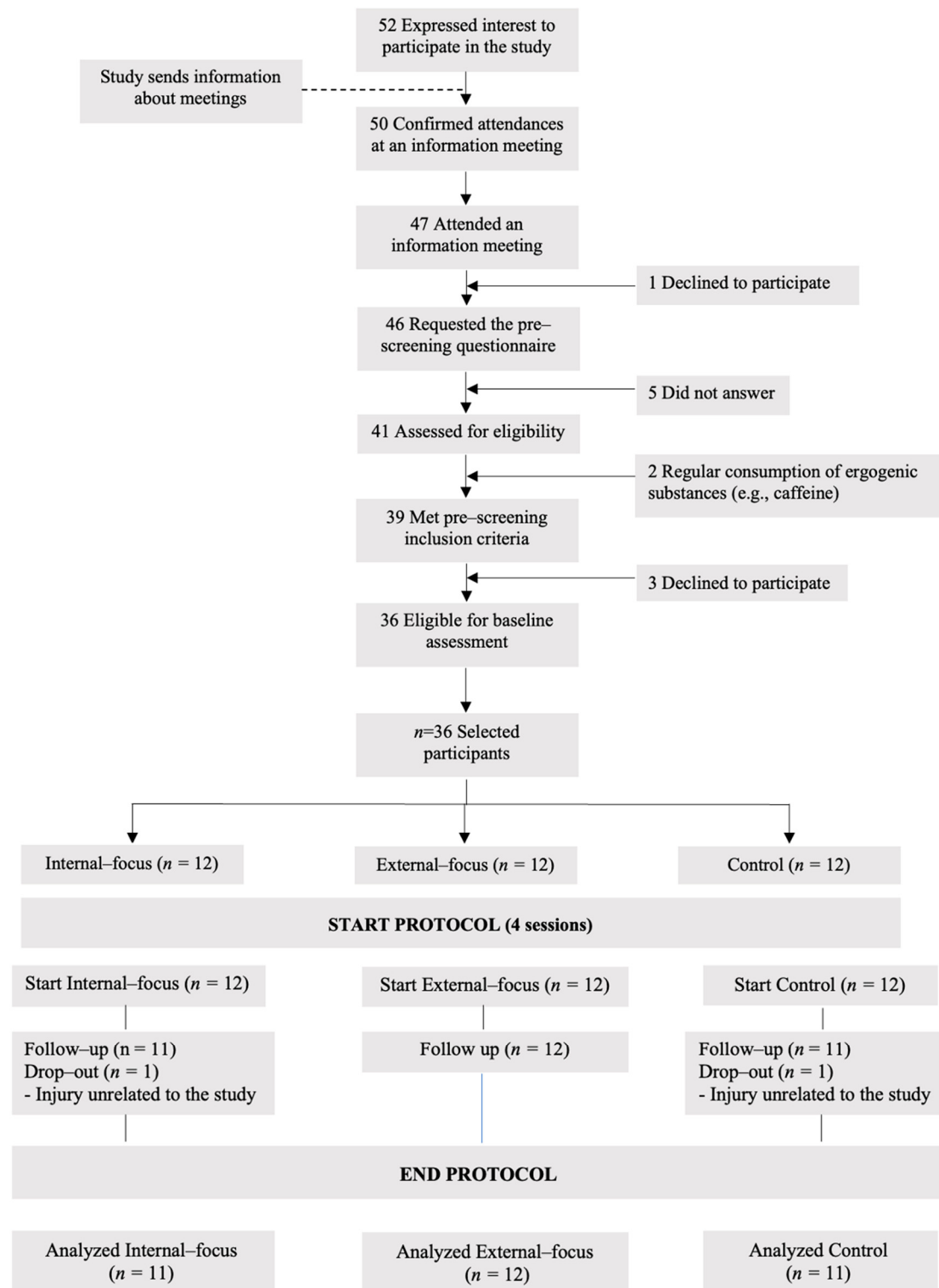

**Figure S1.** Participants flow-chart.

Supplement: Supplementary file 1 [file jfmk-11-00189-s001.zip › jfmk-4246145-supplementary file.pdf]
